# Deletion of the *Prdm3* Gene Causes a Neuronal Differentiation Deficiency in P19 Cells

**DOI:** 10.3390/ijms21197192

**Published:** 2020-09-29

**Authors:** Paweł Leszczyński, Magdalena Śmiech, Aamir Salam Teeli, Effi Haque, Robert Viger, Hidesato Ogawa, Mariusz Pierzchała, Hiroaki Taniguchi

**Affiliations:** 1 Institute of Genetics and Animal Biotechnology, Laboratory for Genome Editing and Transcriptional Regulation, Polish Academy of Sciences, 05-552 Jastrzębiec, Poland; p.leszczynski@ighz.pl (P.L.); m.smiech@ighz.pl (M.Ś.); teeliaamir7@gmail.com (A.S.T.); e.haque@ighz.pl (E.H.); 2 Reproduction, Mother and Child Health, Centre de Recherche du CHU de Québec-Université Laval and Centre de Recherche en Reproduction, Développement et Santé Intergénérationnelle (CRDSI), Quebec, QC GIV4G2, Canada; robert.viger@crchudequebec.ulaval.ca; 3 Department of Obstetrics, Gynecology, and Reproduction, Université Laval, Quebec, QC G1V0A6, Canada; 4 Graduate School of Frontier Biosciences, Osaka University, 1-3 Yamadaoka, Suita 565-0871, Japan; hidesato@fbs.osaka-u.ac.jp; 5 Institute of Genetics and Animal Biotechnology, Department of Genomics and Biodiversity, Polish Academy of Sciences, 05-552 Jastrzębiec, Poland; m.pierzchala@ighz.pl

**Keywords:** P19 cells, Prdm3, Gata6, retinoic acid, neurogenesis, CRISPR, gene knockout

## Abstract

PRDM (PRDI-BF1 (positive regulatory domain I-binding factor 1) and RIZ1 (retinoblastoma protein-interacting zinc finger gene 1) homologous domain-containing) transcription factors are a group of proteins that have a significant impact on organ development. In our study, we assessed the role of *Prdm3* in neurogenesis and the mechanisms regulating its expression. We found that *Prdm3* mRNA expression was induced during neurogenesis and that *Prdm3* gene knockout caused premature neuronal differentiation of the P19 cells and enhanced the growth of non-neuronal cells. Interestingly, we found that *Gata6* expression was also significantly upregulated during neurogenesis. We further studied the regulatory mechanism of *Prdm3* expression. To determine the role of GATA6 in the regulation of *Prdm3* mRNA expression, we used a luciferase-based reporter assay and found that *Gata6* overexpression significantly increased the activity of the *Prdm3* promoter. Finally, the combination of retinoic acid receptors *α* and *β*, along with *Gata6* overexpression, further increased the activity of the luciferase reporter. Taken together, our results suggest that in the P19 cells, PRDM3 contributed to neurogenesis and its expression was stimulated by the synergism between GATA6 and the retinoic acid signaling pathway.

## 1. Introduction

PRDM3 is a transcription factor belonging to the PRDM (PRDI-BF1 (positive regulatory domain I-binding factor 1) and RIZ1 (retinoblastoma protein-interacting zinc finger gene 1) homologous domain-containing) protein family. A characteristic feature of this group of proteins is the PR-SET domain at the N-terminus, the zinc finger C_2_H_2_ motif group, and the acidic domain at the C-terminus [1,2,3]. Mice embryos lacking the *Prdm3* gene exhibit disturbed cardiovascular and nervous system development and die at 10.5 days post coitum [4]. In adult tissues, *Prdm3* is expressed in the lungs, ovaries, kidneys, and brain structures [5,6]. The Hamlet protein (*Drosophila melanogaster* homolog of both PRDM3 and PRDM16) belongs to the molecular machinery involved in the cell fate determination of external sensory organs [7,8] and olfactory receptor neuron diversification [9]. EGL-43 (the PRDM3 homolog in *Caenorhabditis elegans*) is required for the migration of hermaphrodite-specific motor neurons in nematodes [10]. Interestingly, ectopic overexpression of *Prdm3* results in neuronal differentiation [11]. In primary neurons, *Prdm3* mediates transcriptional suppression via histone deacetylase 1 (HDAC1 deacetylase) and controls the synaptic plasticity by inhibiting miR-124 expression [12]. However, despite the importance of PRDM3 in determining the cellular identity [7,13], a clear understanding of the mechanism controlling its expression during neuronal differentiation has yet to be documented.

A possible candidate mechanism that may control *Prdm3* expression is the retinoic acid (RA) signaling pathway. RA is known to be important in the induction of progenitor cell maturation toward neuronal identity [14,15]. RA governs molecular signaling during early neurogenesis in developing brain structures [16,17,18]. In this regard, several studies have demonstrated that *Prdm3* expression is induced during neurogenesis [11,19,20]. Interestingly, it has been reported that crosstalk between GATA proteins and RA signaling exhibits a significant impact on body development [21], suggesting that there are synergistic roles for the GATA factors and RA signaling in cellular differentiation. The GATA family of zinc finger proteins are transcription factors that bind to the WGATAR consensus nucleotide motifs in the regulatory regions of several target genes, and therefore modulate the target gene expression [22]. The role of GATA factors in the development of organs of endodermal origin has been well studied [23]. However, research on their neuronal-specific function remains incomplete. Nevertheless, GATA2 and GATA3 were reported to be involved in the glutamatergic and serotonergic development of the neuronal subtype [24].

In the present study, we investigated the role of PRDM3 in neurogenesis based on the differentiation of P19 cells [25]. Undifferentiated P19 cells can differentiate into neurons through RA stimulation during cell aggregation [25,26]. Hence, P19 cells are commonly used as a model for studying the genetic mechanisms of neuronal development [27,28,29]. We observed a significant increase in *Prdm3* expression during RA-induced cell differentiation. To assess the effect of PRDM3 on neurogenesis, we generated a *Prdm3* knockout (KO) in P19 cells. P19 cells lacking PRDM3 displayed earlier neuronal maturation with a rapid proliferation of non-neuronal cells. Additionally, we identified that RA-dependent signaling and *Gata6* overexpression synergistically increased the activity of the *Prdm3* promoter. Importantly, this study has initiated new directions for the further exploration of PRMD3-dependent mechanisms in neurogenesis.

## 2. Results

### 2.1. The Neural Differentiation of P19 Cells Was Accompanied by an Increased Expression of Prdm3

**Figure 1:**
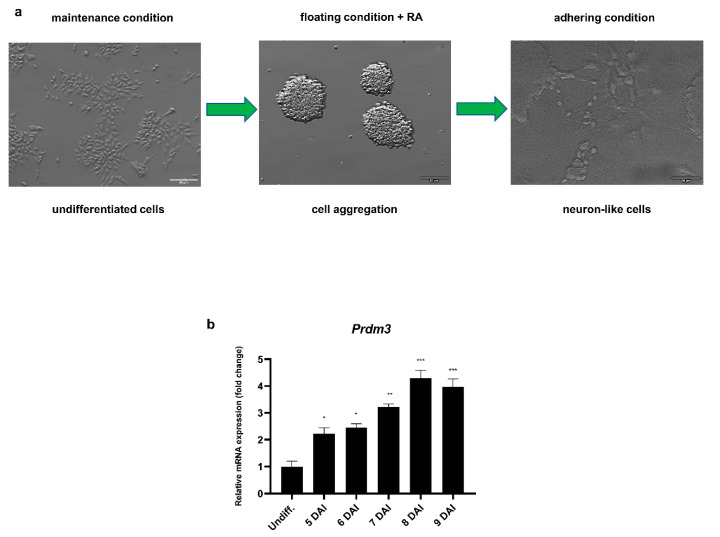
*Prdm3* gene expression was increased during the neurogenesis. (**a**) The undifferentiated P19 cell morphology (left panel), generation of the cell aggregates in a nonadherent dish in the presence of retinoic acid for 4 days (middle panel), and the neuronally differentiated P19 cells 9 days after induction (DAI) with retinoic acid (RA) following cell plating (right panel). The scale bar represents 50 μm. (**b**) The RT-qPCR analysis of the *Prdm3* mRNA expression during neurogenesis. The control group consisted of undifferentiated P19 cells (Undiff.), which were compared with the treated cells 5–9 DAI. *Gapdh* was used as the reference gene. The error bars are shown as the mean ± standard error of the mean (S.E.M.), *n* = 3 per group. * *p* < 0.05, ** *p* < 0.01, and *** *p* < 0.001.

To investigate the role of PRDM3 during the neuronal differentiation, we utilized RA-induced P19 embryonic carcinoma stem cells. Neuronally differentiated P19 cells are morphologically and functionally similar to primary neurons. Hence, the cells are considered to be a convenient and simple model for studying the molecular mechanisms orchestrating neurogenesis [25]. Fully developed neuron-like cells were present 9 days after induction (DAI) with RA following cell plating (Figure 1a). *Prdm3* expression was upregulated during the RA-induced neurogenesis using the Northern blot method [11]. Therefore, we decided to determine the exact expression profile with a greater threshold sensitivity using RT-qPCR. During neuronal differentiation, *Prdm3* was continuously expressed, suggesting a significant role in the transition from a pluripotent state to mature postmitotic neurons (Figure 1b).

### 2.2. Generation of the Prdm3 Knockout P19 Cells

**Figure 2:**
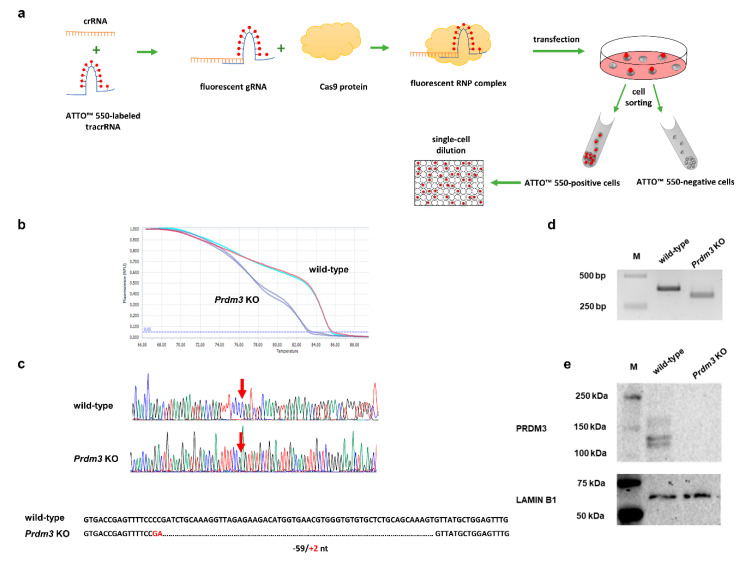
Workflow for the identification of the clustered, regularly interspaced short palindromic repeats/CRISPR-associated protein 9 (CRISPR/Cas9)-mediated *Prdm3* gene deletion in the P19 cells. (**a**) Graphic illustration of the CRISPR/Cas9 genome editing approach for the enrichment and propagation of the cell clones. (**b**) High-resolution melting (HRM) curve analysis of the wild-type (control) and CRISPR/Cas9-edited P19 cells. The curve shift indicates that there were deletions in the CRISPR/Cas9-edited genome sequence. (**c**) A comparison of the sequencing results confirmed that the genome editing was efficient. The edited cells displayed a 59 bp deletion and a 2 bp insertion in the exon 4 sequence compared to the wild-type cells. (**d**) The gel image indicated the faster migration of the shorter amplicon size (*Prdm3* KO) relative to the wild-type cells on a 3% agarose gel. M: DNA-sized ladder. (**e**) The absence of the PRDM3 (PRDI-BF1 (positive regulatory domain I-binding factor 1) and RIZ1 (retinoblastoma protein-interacting zinc finger gene 1) homologous domain-containing transcription factor 3) protein in the *Prdm3* KO cells was confirmed using Western blotting. LAMIN B1 was used as a loading control.

To assess the role of *Prdm3* in neuronal differentiation, we used the clustered, regularly interspaced short palindromic repeats/CRISPR-associated protein 9 (CRISPR/Cas9) method to generate P19 cells that had a disrupted *Prdm3* gene. Our workflow for the CRISPR/Cas9-mediated generation of a cell clone with the desired *Prdm3* mutation is illustrated in Figure 2a. We designed our experiment to target exon 4 of the *Prdm3* gene. Disruption of the gene sequence in this location is sufficient to generate mice with a PRDM3 deficiency [30]. Using the lipotransfection method, the Cas9 protein, along with the crRNA:tracrRNA-ATTO-550 (ribonucleoprotein complex, RNP), were delivered to the undifferentiated P19 cells. Next, we enriched the cell population with an ATTO-550-positive signal via fluorescence-activated cell sorting (FACS). Fluorescent-positive P19 cells were expanded as single-cell clones. After 14 days, only the individual colonies were selected for DNA extraction, followed by high-resolution melting (HRM) curve analysis. The HRM curve analysis enabled us to discriminate between the wild-type (control) and *Prdm3* KO cell clones. A rapid assessment allowed us to choose samples with a highly diverse profile compared to the wild-type DNA (Figure 2b). To confirm the deletion, we sequenced selected samples. A cell clone named P19_C5 (a *Prdm3* KO) showed a 59 bp deletion with a 2 bp insertion in the *Prdm3* gene sequence (Figure 2c). The targeted *Prdm3* gene region was also amplified using PCR and validated using agarose gel electrophoresis. The gel image indicated the faster migration of the shorter amplicon (*Prdm3* KO) relative to the wild-type cells on a 3% agarose gel (Figure 2d). Additionally, we evaluated the PRDM3 protein levels using Western blotting. The result indicated that PRDM3 was not present in the *Prdm3* KO cells, whereas the wild-type cells exhibited an abundance of PRDM3 proteins (Figure 2e).

Finally, we assessed whether the P19 cells carrying the *Prdm3* knockout had any undesirable mutations in the genome (off-targets). Using Sanger sequencing, we evaluated the potential genome sites predicted by CHOPCHOP v2, where the binding RNP complex was designed to generate *Prdm3* KO cells with a high probability. We did not observe any off-target mutations in the *Prdm3* KO P19 cells (Figure S1).

### 2.3. The Prdm3 Gene Disruption Interfered with Neurogenesis in the RA-Induced P19 Cells

**Figure 3:**
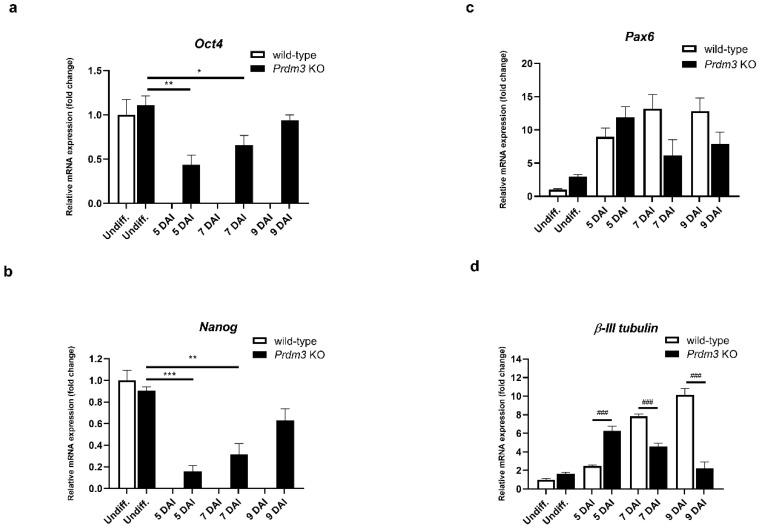
*Prdm3* knockout impeded the RA-induced neurogenesis in the P19 cells, as seen via the qRT-PCR analysis of the (**a**) *Oct4*, (**b**) *Nanog*, (**c**) *Pax6*, and (**d**) *β-III tubulin* mRNA levels during the neurogenesis in the PRDM3-deficient versus wild-type P19 cells. The undifferentiated P19 cells represent the control group and the 5–9 DAI labels signify the days following the cell plating and the RA induction. *Gapdh* was used as the reference gene. The error bars are shown as the mean ± S.E.M., *n* = 3 per group. * *p* < 0.05, ** *p* < 0.01, and *** *p* < 0.001 versus the undifferentiated cells, and ### *p* < 0.001 compared to the wild-type cells within the individual DAI.

Next, we sought to evaluate the role of PRDM3 in neuronal differentiation. First, we investigated the expression of the pluripotency markers *Oct3/4* and *Nanog* using RT-qPCR. Total RNA was isolated from the CRISPR-edited P19 cells and wild-type cells. Undifferentiated P19 cells (Undiff.) expressed stem cell markers (*Oct4* and *Nanog*) at a similar level in the control and *Prdm3* KO cells. Although *Oct4* and *Nanog* expressions were absent in the control cells during neuronal differentiation, the *Prdm3* KO cells exhibited a significant upregulation of *Oct4* and *Nanog* at 5, 7, and 9 DAI. This observation suggested the high proliferation of non-neuronal cells in the *Prdm3* KO cells (Figure 3a,b). We analyzed the impact of the PRDM3 deficiency on the expression of known neuronal marker genes [31]. The *Pax6* gene is involved in the proliferation of radial glial progenitor cells and the migration of immature neurons [32]. We found that *Prdm3* KO cells displayed a downregulation of the *Pax6* mRNA level at 7 and 9 DAI compared to the wild-type cells, but this was not statistically significant (Figure 3c). High *Oct4* and *Nanog* expressions after the RA stimulation suggested that there was a disturbance in the neurogenesis in the *Prdm3* KO cells. To evaluate this observation, we assessed the expression profile of *β-III tubulin* (a neuronal-specific marker) [33]. The analysis revealed that the PRDM3-deficient P19 cells expressed a higher *β-III tubulin* level at 5 DAI compared to the wild-type cells. Surprisingly, in the succeeding days of the neuronal differentiation, the *Prdm3* KO cells displayed a large decrease in their *β-III tubulin* expression, while a gradual upregulation was observed in the wild-type cells (Figure 3d), suggesting that the P19 cells lacking PRDM3 entered into a phase of neuronal maturation earlier than the wild-type cells, though further development was arrested. 

**Figure 4:**
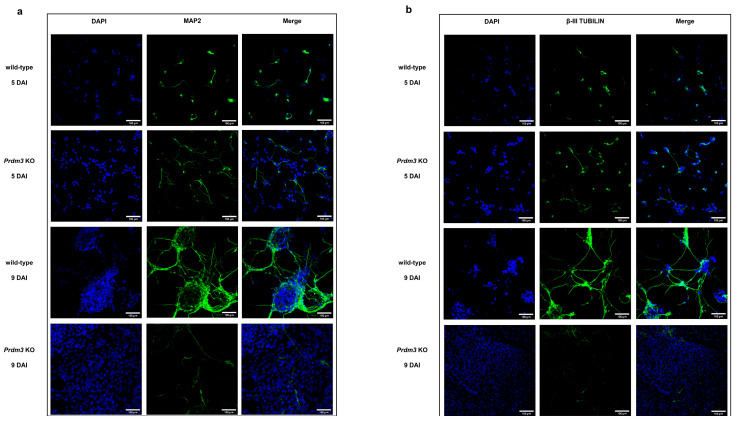
Immunofluorescence microscopy images demonstrating the difference in the microtubule-associated protein 2 (MAP2) and β-III TUBULIN detection in PRDM3-deficient and wild-type P19 cells during neuronal maturation. (**a**,**b**) The MAP2 and β-III TUBULIN staining (green) showed that the *Prdm3* KO P19 cells were relatively well differentiated at 5 DAI, while the MAP2 and β-III TUBULIN in the wild-type cells were barely detectable. On the final day of the RA induction (9 DAI), an overrepresentation of MAP2- and β-III-TUBULIN-negative cells was observed in the PRDM3-deficient group compared to that in the highly MAP2- and β-III TUBULIN-stained wild-type cells. The cell nuclei were stained with 4’,6-diamidino-2-phenylindole (DAPI; blue). The scale bar represents 100 μm.

Additionally, we validated a cytoskeleton protein specific for mature neurons, microtubule-associated protein 2 (MAP2) [34], and β-III TUBULIN [33] using immunofluorescence staining (Figure 4). The PRDM3-deficient cells were highly MAP2- and β-III TUBULIN-positive at 5 DAI compared to the control cells, but they were barely detectable at 9 DAI. Conversely, the wild-type cells displayed enhanced MAP2 and β-III TUBULIN protein expression with a well-defined neuronal morphology at 9 DAI (Figure 4a,b).

### 2.4. GATA Transcription Factors and Retinoic Acid Exerted a Synergistic Effect on the Activity of the Prdm3 Promoter

**Figure 5:**
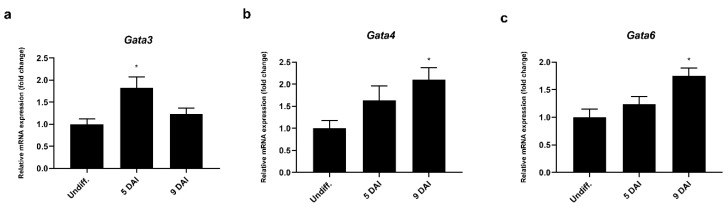
The *Gata3*, *Gata4*, and *Gata6* mRNA expressions were upregulated during the neurogenesis in the P19 cells, as seen in the RT-qPCR analysis of the (**a**) *Gata3*, (**b**) *Gata4*, and (**c**) *Gata6* mRNA expressions. *Gapdh* was used as the reference gene. The control group is abbreviated as Undiff., which represents the gene expression in undifferentiated P19 cells; 5–9 DAI represents the days after the RA induction. The error bars are shown as the mean ± S.E.M., *n* = 3 per group, * *p* < 0.05.

To explore the possible regulatory mechanism involved in *Prdm3* gene expression during neurogenesis, we used the Eukaryotic Promoter Database (EPD) [35] to identify a *Prdm3*-promoting region. The software identified a sequence (1700 bp) that potentially controlled *Prdm3* expression, which was located in the 5’ upstream region from noncoding exon 1. We focused on the potential role played by the GATA proteins due to the presence of multiple GATA-binding consensus sequences within the *Prdm3* promoter. To identify which GATA factors were involved in the regulation of the *Prdm3* expression, we first evaluated the expression profiles of *Gata1*, *Gata3*, *Gata4*, and *Gata6* in the P19 cells during neurogenesis. The mRNA for *Gata3* showed a transitory increase at 5 DAI (Figure 5a), while *Gata4* (Figure 5b) and *Gata6* (Figure 5c) were significantly upregulated at 9 DAI during neurogenesis. We were unable to detect *Gata1* mRNA at any stage of the cell differentiation (data not shown). 

**Figure 6:**
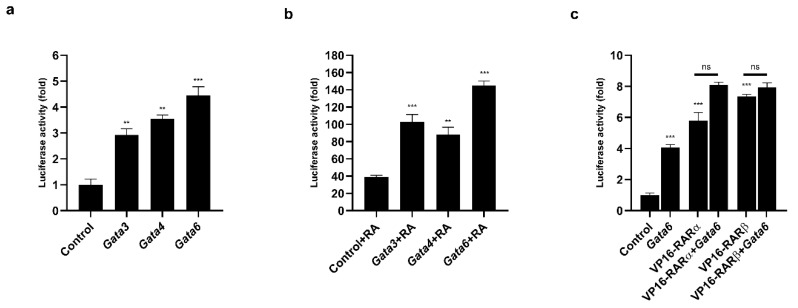
The GATA factors and RA synergistically regulated the *Prdm3* promoter. (**a**) The GATA factors stimulated the activity of the *Prdm3* promoter. The cells were transfected with a plasmid encoding a luciferase reporter gene that was driven by the *Prdm3* promoter with expression plasmids for either *Gata1*, *Gata3*, *Gata4*, *Gata6*, or an empty plasmid (pcDNA3) as a control. (**b**) The RA and GATA factors synergistically activated the *Prdm3* promoter. The P19 cells were cotransfected with pcDNA3 or *Gata* expression vectors, along with the *Prdm3* promoter fused to the luciferase reporter. The cells were cultured with RA (0.5 µM) for 24 h. (**c**) The effect of the retinoic acid receptors (RARs) on the *Prdm3* promoter activity. The P19 cells were transfected with the *Prdm3* promoter, along with pcDNA3, or with *RARα*, *RARβ*, and *Gata6* expression vectors that were used alone or in combination. The luciferase activity is shown as a fold change. Error bars are shown as the mean ± S.E.M., *n* = 3 per group. ** *p* < 0.01, *** *p* < 0.001, and ns (not statistically significant).

We identified several consensus binding sites for the GATA proteins (Figure S2). To determine whether the GATA factors modulated the activity of the *Prdm3* promoter, we generated a plasmid carrying a luciferase reporter gene under the control of the *Prdm3* promoter. As shown in Figure 6a, the overexpression of the GATA factors increased the activity of the *Prdm3* promoter more than 3-fold. However, the strongest effect was observed for the *Gata6* overexpression (approximately 4.5-fold) (Figure 6a). To determine the mechanism by which GATA proteins influenced the activity of the *Prdm3* promoter, we used a truncated GATA4 protein construct (a dominant-negative competitor, DN), consisting solely of its zinc finger DNA-binding domain [36]. Since the DN protein is devoid of its transactivation domains, it can bind to, but not activate, GATA-dependent target promoters [36]. The structure of the DN construct is based on the GATA4 sequence, but due to the high conservation of the zinc finger domains within the GATA protein family, it can be used as a general competitor for GATA binding [37]. In our study, the DN construct was used to examine the contribution of GATA6 proteins to the activity of the *Prdm3* promoter in P19 cells. We cotransfected the *Gata6* plasmid along with increasing doses of the DN construct (50, 150, and 300 ng). The DN competitor significantly decreased the promoter activity in a dose-independent manner but did not stop the promoter activity (Figure S3). This indicated that the *Prdm3* promoter may have been partially dependent on the direct binding of GATA proteins to the GATA consensus elements found in the *Prdm3* promoter. To assess the potential functional relationship between GATA factors and RA in the neurogenesis, we first tested the effect of RA stimulation on the activity of the *Prdm3* promoter. Compared to the control cells, a concentration of 0.5 µM RA (control + RA) increased the luciferase activity by approximately 40-fold (Figure 6b). Next, we investigated whether the selected GATA factors and the RA treatment could synergistically activate the *Prdm3* promoter. For this purpose, the P19 cells were co-transfected with a control plasmid, along with plasmids encoding *Gata1*, *Gata3*, *Gata4*, or *Gata6*. After one day post-transfection, the same set of cells was treated with RA (0.5 µM) for 24 h and then harvested for a luciferase assay. The combination of the RA stimulation and overexpression of the GATA factors led to a strong synergistic upregulation in the activity of the *Prdm3* promoter. The most pronounced synergism was observed with the combination of *Gata6* and RA treatment, where the increase in the luciferase activity was approximately 140-fold (Figure 6b). To better understand the molecular basis of the RA-dependent stimulation, we focused on the RA receptors (RARs). RARs mediate the molecular effects of RA by interacting with the retinoic acid response elements (RAREs) in the gene regulatory regions [38]. It was also reported that RA stimulates *RARα* and *RARβ* mRNA expression in P19 cells [39,40]. Therefore, we tested the effect of pCMX-VP16-*RARα* and pCMX-VP16-*RARβ* on the activity of the *Prdm3* promoter. VP16-*RAR* is a constitutively active form of the RA receptor that is fused to a VP16 activation domain. In the luciferase assay, the overexpression of VP16-*RARα* and VP16-*RARβ* triggered a significant increase in the activity of the *Prdm3* promoter (Figure 6c). The most potent activation was observed in the case of the VP16-*RARβ* overexpression. Next, in an attempt to explain the synergistic effect between the GATA factors and the RA stimulation, we validated the additive effects of RARα/RARβ with GATA6 on the activity of the *Prdm3* promoter. Cotransfection of both plasmids showed a large increase in luciferase activity, but the result was not synergistic (Figure 6c). Instead, the observed effect showed an additive tendency regarding the combination of RARα or RARβ with GATA6. These data suggest that the *Prdm3* expression could be partially under the control of RARs, but the molecular mechanism underlying the synergistic effect of the GATA factors and the RA stimulation remains to be defined. 

Finally, we identified an inverted RARE consensus sequence (CGACCT*ttttg*TGACCT) within the *Prdm3* promoter, where the RAREs consisted of two sequence domains separated by five nucleotides (marked in italics) [41]. We mutated each domain separately to assess the importance of this element in the RARα- and RARβ-dependent activation of the *Prdm3* promoter. We observed that each mutation of the RARE domain did not eliminate the *Prdm3* promoter activity after the RARs’ stimulation (Figure S4). This suggested that the RARs regulated, at least in part, the *Prdm3* promoter activity through their binding to the RARE domain in the *Prdm3* promoter.

## 3. Discussion

We determined the role of PRDM3 in neuronal differentiation using P19 cells, which is a well-established model that is used to study the early stages of mammalian nervous system development [26,27,28,29]. Disruption of PRDM3 expression has been shown to lead to slow brain and spinal development [4]. A repressive function of PRDM3 in *Drosophila melanogaster* was also suggested, where Hamlet (the *Drosophila* homolog of PRDM3 and PRDM16 in mammals) was shown to have a crucial involvement in sensory neuron differentiation [2]. Although PRDM3 and PRDM16 share a similar structure and probably have analogous functions [42], the number of reports on the role of PRDM16 in the nervous system is constantly increasing [43,44,45,46]. However, the function of PRDM3 remains enigmatic and poorly characterized. Our current findings suggest that PRDM3 was required to maintain normal neurogenesis in mammals. Additionally, we found that GATA factors and activation of the RA-dependent molecular pathway might have been involved in the *Prdm3* gene expression during neuronal differentiation.

During the early development of zebrafish, Prdm3 proteins are expressed in brain structures [47]. Furthermore, *Prdm3* expression was also reported in adult mouse brains [48], where its expression was found specifically in the postmitotic neurons [12]. Nevertheless, the precise expression pattern during neuronal development is not well known. In our study, we showed that *Prdm3* expression was upregulated during neurogenesis using the P19 cells (Figure 1b). Increased *Prdm3* expression was observed in the global gene expression analysis during the neuronal differentiation of human embryonic cells and mouse P19 cells [19]. These findings collectively point to a possible role for PRDM3 in mammalian neurogenesis. However, this possibility has so far been based only on circumstantial gene expression profiling evidence and not on the direct effect of PRDM3 in neuronal differentiation. For this reason, we generated PRDM3-deficient P19 cells using a CRISPR/Cas9 strategy. Surprisingly, the PRDM3-deficient P19 cells displayed an increased expression of *Oct4* and *Nanog* at 7 and 9 days after the RA induction (Figure 3a,b), suggesting a significant retainment of cell stemness and an impairment of the neurogenic potential of the deficient cells. PAX6 (paired box 6) plays a fundamental role during the early stage of neurogenesis, where it maintains the balance between the self-renewal of neural stem cells and neuronal maturation [31]. Consistent with this function, *Pax6* mRNA levels were upregulated during neurogenesis in wild-type P19 cells, but this upregulation was hindered in the PRDM3-deficient P19 cells during neuronal differentiation (Figure 3c). Interestingly, we found that the expression of mature neuron-specific markers, namely, β-III TUBULIN and MAP2, in the PRDM3-deficient cells was substantially different than that in the control cells (Figure 4a,b). At 5 DAI, *β-III tubulin* was highly upregulated in the modified cells but this effect was lost by 9 DAI (Figure 3d). A similar observation was found for the anti-MAP2 and β-III TUBULIN staining (Figure 4a,b). The gradual loss of the *β-III tubulin* expression in the days following the cell seeding might be explained by the non-neuronal cell overgrowth, where this observation was also consistent with the increased *Oct4* and *Nanog* expression (Figure 3a,b). On the other hand, the expression level of *β-III tubulin* at 5 DAI was significantly higher in the PRDM3-deficient P19 cells than that in the wild-type cells. Thus, the P19 cells lacking PRDM3 appeared to enter neural maturation earlier than the wild-type cells but could not continue this process in the absence of PRDM3. PRDM3 may have a stepwise involvement in the regulation of neurogenesis. Therefore, our data show that during RA stimulation, PRDM3 was part of the molecular machinery controlling the stage-dependent neuronal maturation, at least in the P19 cells.

It has been suggested that GATA2 and GATA3 affect the specialization of neuronal differentiation into glutamatergic and serotonergic subtypes [24]. This suggestion prompted us to evaluate the effect of GATA factors on the regulation of *Prdm3* expression. *Gata3*, *Gata4*, and *Gata6* upregulation were observed in the days following the RA-induced differentiation (Figure 5a–c). Moreover, each of the tested GATA factors stimulated the activity of the *Prdm3* promoter, where the strongest effect was observed with *Gata6* (Figure 6a). It was reported that *Prdm3* expression is stimulated by RA in NTERA-2 human teratocarcinoma cells and acute myeloid leukemia cells [49,50]. In our study, RA increased the activity of the *Prdm3* promoter by approximately 40-fold in luciferase assays, but along with *Gata6* overexpression, we observed a particularly high synergy of both factors (Figure 6b). Moreover, it was reported that there is a functional interaction between an RA-dependent pathway and GATA2 in an embryonic stem cell model of hematopoietic differentiation [21]. These results are consistent with our findings and suggest that there is a close relationship between the *Prdm3* expression, GATA factors, and the RA-dependent molecular pathway. Additionally, we assessed whether the involvement of GATA proteins with the *Prdm3* promoter was the result of direct GATA binding. The *Prdm3* promoter nucleotide sequence contains multiple potential GATA-binding consensus motifs, which makes assessing the individual motifs challenging. To overcome this problem, we used a truncated GATA4 protein, which acted as a DN for all GATA proteins [36]. The GATA DN significantly decreased, but did not stop, the GATA6-induced *Prdm3* promoter activity. This suggested the influence of GATA proteins on Prdm3 expression during neurogenesis. However, the effect of the GATA proteins could have been mediated by the regulation of other transcriptional factors or by affecting the differentiation state of the neurons. Accordingly, further studies are needed to elucidate how GATA proteins regulate *Prdm3* expression during neurogenesis. Taken together, our results provide an additional functional example of the close relationship between *Prdm3* expression, GATA factors, and RA-induced molecular signaling. RA stimulates a vast number of molecular pathways [51,52], which can coincide with GATA factors during neuronal differentiation, and thus, might orchestrate *Prdm3* expression.

We also tested whether *Prdm3* expression could be regulated through the interaction of RARs and GATA proteins. RARs are activated by RA binding and act as transcription factors that contribute to the diversification of the neuronal cell types [53,54,55]. We found a RARE within the *Prdm3* promoter. We confirmed that the RARs stimulated the *Prdm3* promoter activity through the binding of the RARs to the RARE (Figure S4). Moreover, the functional synergism between GATA2 and RARα through protein–protein interaction was demonstrated in human KG1 myeloid cells [21]. In our study, the overexpression of *Gata6* and the *RAR*s was only additive and did not exceed the effect observed with the RA-dependent stimulation (Figure 6c). Thus, it appears that the synergism between GATA- and RA-dependent pathways could only be partially explained by the participation of the RARs. RA is well recognized for its ability to promote neuronal development in embryos [56], but many of the molecular targets remain largely unidentified. Even less is known about the GATAs’ downstream targets in mouse and human nervous systems [57]. In our study, we could not determine the exact molecular mechanism underlying the synergism effect between the RA signaling and the GATA factors observed in the P19 cells. As such, we could not exclude the influence of other factors on the activity of the *Prdm3* promoter, especially those whose expression is stimulated by RA and/or GATA factors. Further studies are needed to clarify this point. 

## 4. Materials and Methods 

### 4.1. Cell Culture

The P19 cells (donated by Katsuhiko Mikoshiba, RIKEN Center for Brain Science, Wako, Japan) were cultured in Dulbecco’s modified Eagle medium (DMEM) supplemented with 4.5 g/L of glucose (Lonza, Basel, Switzerland), 10% of fetal bovine serum (FBS) (EURx, Gdansk, Poland), 100 units/mL of penicillin, and 100 units/mL of streptomycin (Lonza, Basel, Switzerland). The cells were maintained in an incubator at 37 °C and 5% CO_2_. Briefly, the P19 cells (1 × 10^6^ cells) were cultured on a 10 cm bacterial-grade Petri dish (Corning, Corning, NY, USA) in media containing DMEM/5% FBS and 0.5 µM all-*trans* retinoic acid (Sigma, St. Louis, MO, USA) for 4 days. During this period, the cells aggregated and resembled the structure of an embryonic body (EB). To induce neuronal differentiation, the P19 cells were trypsinized and plated into adherent culture dishes in DMEM/10% FBS for the next 5 days [25].

### 4.2. Quantitative RT-PCR (RT-qPCR)

Total RNA was isolated from cultured cells using a fenozol reagent (A&A Biotechnology, Gdynia, Poland). For the reverse transcription, 0.5 μg of total RNA was used and the reactions were performed according to the manufacturer’s instructions (EURx, Gdansk, Poland). Next, the RT-qPCR was performed with using a Fast SG qPCR Master Mix (EURx, Gdansk, Poland), and the gene expression was examined using a LightCycler 96 Instrument (Roche, Mannheim, Germany) under the following conditions: incubation at 95 °C for 5 min, followed by 40 cycles of 95 °C for 10 s, 60 °C for 10 s, and 72 °C for 10 s. The expression of the target gene was normalized against glyceraldehyde-3-phosphate dehydrogenase (*Gapdh*). The sequences of primers used for the RT-qPCR are presented in Table 1.

### 4.3. Generation of the Prdm3 Gene-Inactivated P19 Cells

The PRDM3-deficient P19 cells were generated using the CRISPR/Cas9-mediated gene editing method. The sequence of crRNA (5′-TCTCTAACCTTTGCAGATCG-3′) was designed to target the mouse *Prdm3* gene in the location of exon 4 using CHOPCHOP v2 [58]. Selected crRNA and ATTO-550-labeled tracrRNA were purchased from Integrated DNA Technologies (Coralville, IA, USA). Briefly, the cells were plated 24 h before transfection (3.5 × 10^4^ cells/well in 24-well plates). A duplex of crRNA and tracrRNA-ATTO-550 was prepared in equimolar concentrations (1 μM), heated at 95 °C for 5 min, and cooled down to room temperature (RT). The formation of the ribonucleoprotein (RNP) complex was generated via the assembly of 1 μM of Cas9 protein (Integrated DNA Technologies, Coralville, IA, USA) with a crRNA:tracrRNA-ATTO-550 duplex in Opti-MEM (Thermo Fisher Scientific, Waltham, MA, USA) and incubated for 10 min at RT. Next, 1.5 µL of Lipofectamine CRISPRMAX (Thermo Fisher Scientific, Waltham, MA, USA) was added to the RNP complex, mixed, and incubated at RT for 10–15 min. Finally, the mixture was added dropwise onto the cell medium. To address the potential off-target effects of the CRISPR/Cas9 method, we used the CHOPCHOP v2 program to select sites in the genome that had a high sequence similarity to the crRNA binding site in the *Prdm3* gene [58]. We designed a primer set to amplify the potential off-target regions (Table S1) and performed PCR amplification. Next, we verified the selected fragments using Sanger sequencing (Genomed, Warsaw, Poland) (Figure S1).

### 4.4. Western Blotting

The nuclear protein concentrations from the wild-type and PRDM3-deficient P19 cells were determined using the Pierce BCA Protein Assay Kit (Thermo Fisher Scientific, Waltham, MA, USA). Protein extracts (20 µg) were separated on 4–15% precast gel (Bio-Rad, Hercules, CA, USA) and transferred to a PVDF membrane (Merck Millipore, Burlington, MA, USA) using a wet transfer. Blots were probed overnight with a primary antibody for PRDM3 (1:1000, Cell Signaling Technology, Danvers, MA, USA) in 5% skim milk in TBS (tris-buffered saline, Sigma-Aldrich, Saint Louis, MO, USA) supplemented with 0.1% tween-20 (Sigma-Aldrich, Saint Louis, MO, USA), followed by incubation with HRP-conjugated anti-rabbit IgG produced in goats (1:5000, Sigma-Aldrich, Saint Louis, MO, USA) in 5% skim milk in TBST for 1 h at RT. Proteins were visualized using the ECL Western Blotting Analysis System (Amersham, Illinois, CA, USA) and ChemiDoc XRS+ System (Bio-Rad, Hercules, CA, USA). The molecular weight of the proteins was estimated using the Precision Plus Protein WesternC Standards (Bio-Rad, Hercules, CA, USA). Lamin B1 (1:1000, Santa Cruz Biotechnology, Dallas, TX, USA) was used as a loading control for the Western blotting.

### 4.5. FACS Analysis

After the transfection (24 h), the P19 cells labeled with tracrRNA-ATTO-550 (Integrated DNA Technologies, Coralville, IA, USA) were isolated using fluorescence-activated cell sorting (FACS). Cells treated with a fluorescence RNP complex, but without a transfection reagent, were used as a control to set the gate during the cell sorting. The flow cytometric analysis was performed on a FACSAria (BD Biosciences, San Jose, CA, USA) using Cell Quest software (BD Biosciences, San Jose, CA, USA) in the Department of Immunology at the University of Warsaw, Poland.

### 4.6. Establishment of the Edited P19 Cells from Single-Cell Clones

FACS-sorted P19 cells were diluted to 1 cell/100 µL by adding 100 cells into 10 mL DMEM/10% FBS. Then, 100 µL of the cell suspension was distributed into each well of the 96-well plate and incubated for 1 week. The wells with a visible single-cell colony were washed with 100 µL of PBS (Phosphate Buffered Saline) without calcium and magnesium (Lonza, Basel, Switzerland) and then detached by adding 20 µL of trypsin-EDTA (0.25%) (Biosera, Nuaille, France). Next, the cells were split in the same arrangement into two 96-well plates. After one week, the cells reached an approximately 80% confluence and were used for further analysis.

### 4.7. Genomic DNA Isolation, HRM PCR, and Sanger Sequencing

The genomic DNA was isolated using the Genomic Mini kit (A&A Biotechnology, Gdynia, Poland) following the manufacturer’s instructions. The HRM PCR reaction was prepared by mixing 5 μL of RT HS-PCR Mix EvaGreen (A&A Biotechnology, Gdynia, Poland) 0.25 μL of each primer (10 μM), and 1 μL of isolated DNA (10 ng), and then adjusted with water up to 10 μL. The primers used for the HRM PCR and PCR are presented in Table 2. The HRM PCR reaction was carried out in a LightCycler 96 (Roche, Mannheim, Germany) using the following HRM PCR conditions: 95 °C for 5 min, then 40 cycles of 95 °C for 15 s and 60 °C for 15 s, 72 °C for 20 s, followed by one cycle of 95 °C for 30 s and 60 °C for 60 s. The melting analysis was performed by preheating the PCR product to 95 °C. Then, the mixture was cooled down to 40 °C for 1 min, followed by heating to 65 °C. A continuous fluorescent signal was acquired from 65 to 97 °C. The amplified curves were analyzed using LightCyler 96 software (Roche, Mannheim, Germany). The sequence of the PCR products was assayed using Sanger sequencing (Genomed, Warsaw, Poland).

### 4.8. Plasmid Construction

To amplify the *Prdm3* promoter sequence located in the 5’ region upstream from noncoding exon 1, we isolated the genomic DNA from P19 cells using a Genomic Mini kit (A&A Biotechnology, Gdynia, Poland). The primers for the selected region were designed based on the sequence located on chromosome 3, namely, 30,014,710 to 30,013,010. KpnI and HindIII restriction sites were incorporated into the forward and reverse primers, respectively. The primers used for cloning the *Prdm3* promoter are depicted in Table 3. The *Prdm3* promoter was amplified via PCR using PrimeSTAR Max DNA Polymerase (TaKaRa, Shiga, Japan) according to the manufacturer’s instructions. A pGL3 basic-vector-coding luciferase gene (Promega, Madison, WI, USA) was digested with KpnI and HindIII enzymes (Thermo Fisher Scientific, Waltham, MA, USA) and ligated with a PCR product using the Gibson assembly method. In brief, the purified DNA fragments (1:9 molar ratio of pGL3 vector to the insert) were resuspended in nuclease-free water. An OverLap™ Assembly (A&A Biotechnology, Gdynia, Poland) kit was used for the cloning. The components used were 4 µL of OverLap Assembly Buffer, 2 μL of nucleotides, 2 µL of OverLap™ Assembly Enzyme Mix, and DNA fragments; the components were mixed on ice and adjusted with water to a final volume of 20 µL. The assembly reaction was carried out at 50 °C for 15 min. Positive bacterial clones were selected for the plasmid isolation and then sequenced. VP16-*RARα* and VP16-*RARβ* were cloned into the pCMX plasmid. pcDNA3-*Gata1*, pcDNA3-*Gata3*, pcDNA3-*Gata4*, pcDNA3-*Gata6*, and GATA DN plasmids were reported [36,59]. The RARE site located in the *Prdm3* promoter was mutated using a Site-Directed Mutagenesis Kit (Agilent Technologies, Santa Clara, CA, USA) according to the manufacturer’s instructions. Specific primers for the reaction were designed using the QuikChange Primer Design tool (Agilent Technologies, Santa Clara, CA, USA). Mutated sequences of the receptor binding sites were confirmed using Sanger sequencing. The primer sequences used for the mutagenesis of the RARE site are depicted in Table S2.

### 4.9. Cell Transfection and the Luciferase Assays

For the luciferase assays, 3 × 10^4^ cells were seeded into 24-well plates. After 24 h, the cells were transfected using Lipofectamine 3000 (Thermo Fisher Scientific, Waltham, MA, USA). The cells were harvested after 48 h and the luciferase activity was assayed using a Luciferase Assay Kit (Promega, Madison, WI, USA) according to the enclosed protocol with a Synergy LX luminometer (Biotek, Winooski, VT, USA).

### 4.10. Immunofluorescence

During the RA-induced neurogenesis, the P19 cells were fixed with 4% paraformaldehyde in PBS (Sigma-Aldrich, Saint Louis, MO, USA) for 15 min at RT. The fixed cells were then permeabilized with 0.5% Triton x-100 (Sigma-Aldrich, Saint Louis, MO, USA) for 10 min and incubated for 20 min with non-fat milk (1%) to block the non-specific binding of antibodies. After the blocking, the cells were incubated separately with anti-MAP2 rabbit antibody (Thermo Fisher Scientific, Waltham, MA, USA, diluted 1:200) and β-III TUBULIN (Cell Signaling Technology, Danvers, MA, USA, diluted 1:200) rabbit antibody in 1% non-fat milk overnight at 4 °C. The next day, the cells were washed with 0.1% Triton x-100 in PBS. A goat anti-rabbit IgG secondary antibody, namely, Alexa Fluor Plus 488 (Thermo Fisher Scientific, Waltham, MA, USA, diluted 1:500), was used for the detection of MAP2 and β-III TUBULIN (1 h incubation at RT). The cell nuclei were counterstained with 1 μg/mL of 4′,6-diamidino-2-phenylindole (DAPI) (Thermo Fisher Scientific, Waltham, MA, USA) for 10 min. The cells were finally washed with PBS and mounted on slides with ProLong™ Gold Antifade Mountant (Thermo Fisher Scientific, Waltham, MA, USA). The samples were imaged with a 20× objective using a Nikon A1R confocal microscope (Nikon, Tokyo, Japan). The microscopic pictures were analyzed using Nikon Instruments and ImageJ software (version 1.52q, NIH, Bethesda, MD, USA).

### 4.11. Statistical Analysis

Data are presented as the mean ± standard error of the mean (S.E.M.) of each group in the experiment. The statistical analysis was done using a one-way analysis of variance (ANOVA), followed by Tukey’s post hoc tests. Any *p*-value < 0.05 was considered statistically significant. GraphPad PRISM software version 6 (GraphPad Software, La Jolla, CA, USA) was used for the statistical analysis.

## Figures and Tables

**Table 1:** Primers used for the RT-qPCR.

Gene Name	Primer Sequence	Product Size (bp)
*Gapdh*	F: TGACCTCAACTACATGGTCTACA R: CTTCCCATTCTCGGCCTTG	85
*β-III tubulin*	F: CCCAGCGGCAACTATGTAGG R: CCAGACCGAACACTGTCCA	144
*Oct4*	F: GGCGTTCTCTTTGGAAAGGTGTTC R: CTCGAACCACATCCTTCTCT	313
*Pax6*	F: TAGCCCAGTATAAACGGGAGTG R: CCAGGTTGCGAAGAACTCTG	131
*Prdm3*	F: TTGTTTCACCCGCAATTC R: CGTGTTAGGTTCGCAGACC	235
*Gata3*	F: CCCCATTACCACCTATCCGC R: CCTCGACTTACATCCGAACCC	106
*Gata4*	F: CTCTGGAGGCGAGATGGGAC R: CGCATTGCAAGAGGCCTGGG	254
*Gata6*	F: TTGCTCCGGTAACAGCAGTG R: GTGGTCGCTTGTGTAGAAGGA	105

**Table 2:** Primers used for the high-resolution melting (HRM) PCR and PCR.

Primer Name	Primer Sequence	Product Size (bp)
E4A_HRM_PCR	F: TCTCCGAGAGATCCATGGCA R: TCTTCCCCCGAGCAAACTTG	149
E4A_PCR	F: GGACTTTTGGATCCCACCTT R: GGCCAGTTGTTTTGAAGCTC	375

**Table 3:** Primers used for cloning.

Primer Name	Primer Sequence	Product Size (bp)
Gibson_PCR	F: TTTCTCTATCGATAGGTACCGCCACCAAAATGAATTAGTCACC R: CCGGAATGCCAAGCTTAGCTCCAGGGGCAAGACC	1700

## Supplementary Materials

The following are available online at https://www.mdpi.com/1422-0067/21/19/7192/s1, Figure S1: Sanger sequencing shows no mutations in putative off-target sites, Figure S2: Sequence of *Prdm3* promoter with indicated GATA binding sites and RARE consensus sequence, Figure S3: A GATA dominant negative competitor decreases GATA6-dependent activation of *Prdm3* promoter activity, Figure S4: Effect of a RARE mutation in the Prdm3 promoter on RARs stimulation, Table S1: Putative off-target sites for CRISPR-mediated *Prdm3* gene knock-out, Table S2: Sequence of the primers used for mutagenesis of RARE sites.

## Abbreviations

EB	Embryonic body
Gapdh	Glyceraldehyde-3-phosphate dehydrogenase
CRISPR	Clustered, regularly interspaced short palindromic repeats
Cas9	CRISPR-associated proteins 9
tracrRNA	*Trans*-activating CRISPR RNA
crRNA	CRISPR RNA
FACS	Fluorescence-activated cell sorting
RNP	Ribonucleoprotein
HRM	High-resolution melting curve
PCR	Polymerase chain reaction
MAP2	Microtubule-associated protein 2
Oct4	Octamer-binding transcription factor 4
RARα	Retinoic acid receptor alpha
RARβ	Retinoic acid receptor beta
Pax6	Paired box 6
β-III tubulin	Tubulin beta 3 class III
DAI	Days after induction
GATA6	GATA-binding factor 6
Prdm3	(positive regulatory domain I-binding factor 1) and RIZ1 (retinoblastoma protein-interacting zinc finger gene 1) homologous domain containing transcription factor 3
RA	Retinoic acid
RARE	Retinoic acid response element
