# Supplementary material for: Deletion of the Prdm3 Gene Causes a Neuronal Differentiation Deficiency in P19 Cells
Source: Int J Mol Sci. 2020 Sep 29;21(19):7192. doi: 10.3390/ijms21197192 (PMC7582457; doi:10.3390/ijms21197192)
Supplement: Supplementary file 1 [file ijms-21-07192-s001.pdf]

Supplementary Materials

Table S1. Putative off-target sites for CRISPR-mediated *Prdm3* gene knock-out. Sequence of the primers used to check putative top two off-target sites are indicated below. Mismatched nucleotides in sequence for crRNA binding are marked in red.

Off-target site no.	Target sequence	Number of mismatches	Primer sequence	Product size
1	TCTCTAACCTTTGCAcAggGtGG	4	**F**: GGGACACTTGTGTGCATGAT<br>**R**: GCAAATGCCCTTCCAATCTA	543 bp
2	TCTCTAACtTTTGCAcATCtTGG	3	**F**: TACTTGGTCGCAAGCTGATG<br>**R**: GCCAACTGTCTGCAAGTGAA	629 bp

Table S2. Sequence of the primers used for mutagenesis of RARE sites.

RARE site	Primer sequence
RARE_mut1	**F**: GGCGGGAGAGGAAACAAAAAGGTCGCCAAGACCC<br>**R**: GGGTCTTGGCGACCTTTTTGTTTCCTCTCCCGCC
RARE_mut2	**F**: GGAGAGGTCACAAAAAGGAAGCCAAGACCCAAGTCCTA<br>**R**: TAGGACTTGGGTCTTGGCTTCCTTTTTGTGACCTCTCC

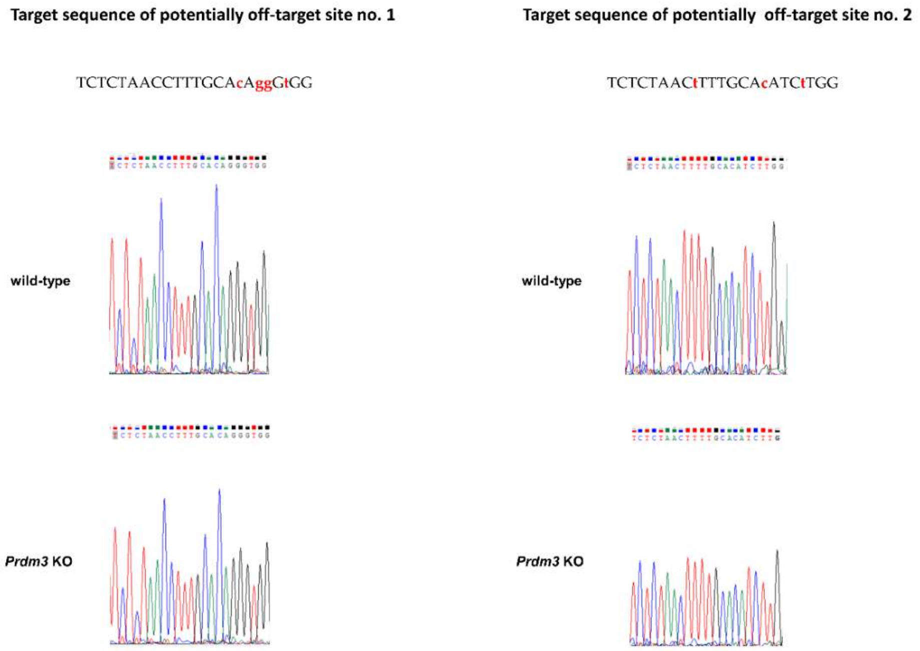

Figure S1. Sanger sequencing shows no mutations in putative off-target sites. Mismatched nucleotides in the crRNA binding sequence are highlighted in red.

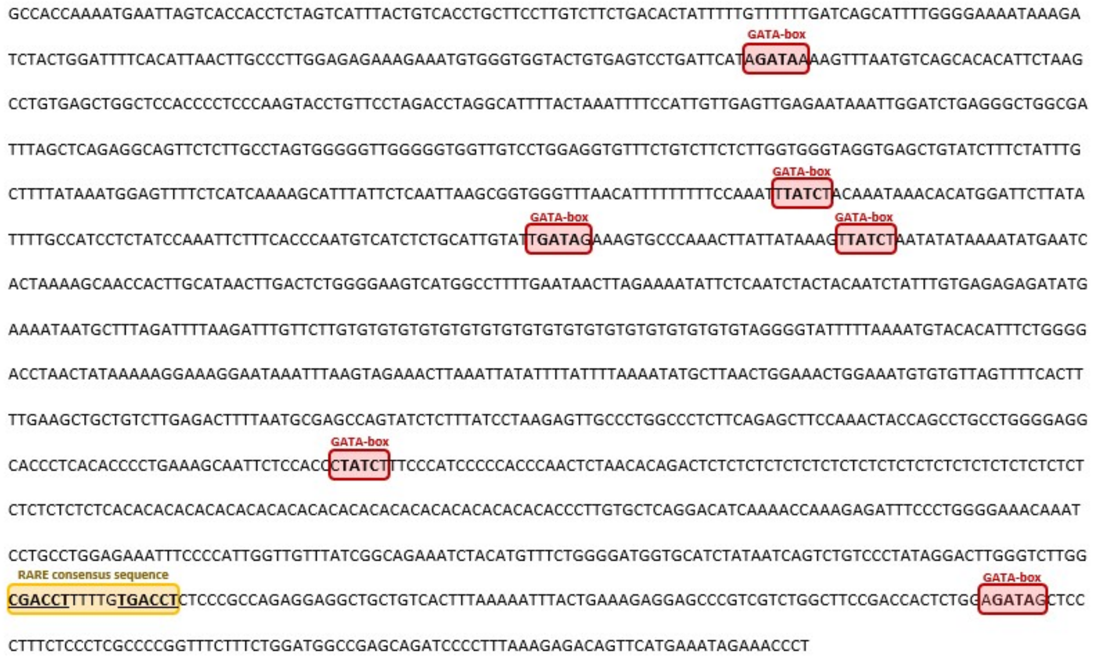

Figure S2. Sequence of *Prdm3* promoter with indicated GATA binding sites and RARE consensus sequence

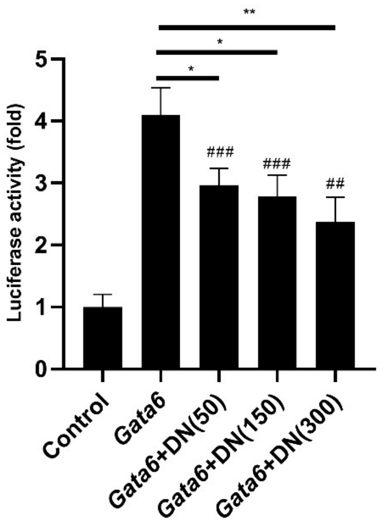

Figure S3. A GATA dominant negative competitor decreases GATA6-dependent activation of *Prdm3* promoter activity. *Prdm3* promoter along with pcDNA3 or GATA6 expression vector in combination with increasing dose of DN (50, 150, 300ng) were transfected in P19 cells. All transfections were performed using a constant amount of DNA (500 ng per culture well). Luciferase activity is shown as a fold change over control. Error bars are shown as mean ± S.E.M., n=4 per group. *p<0.05, **p<0.01, compared to *Gata6* group. ##p<0.01 ### p<0.001 compared to Control.

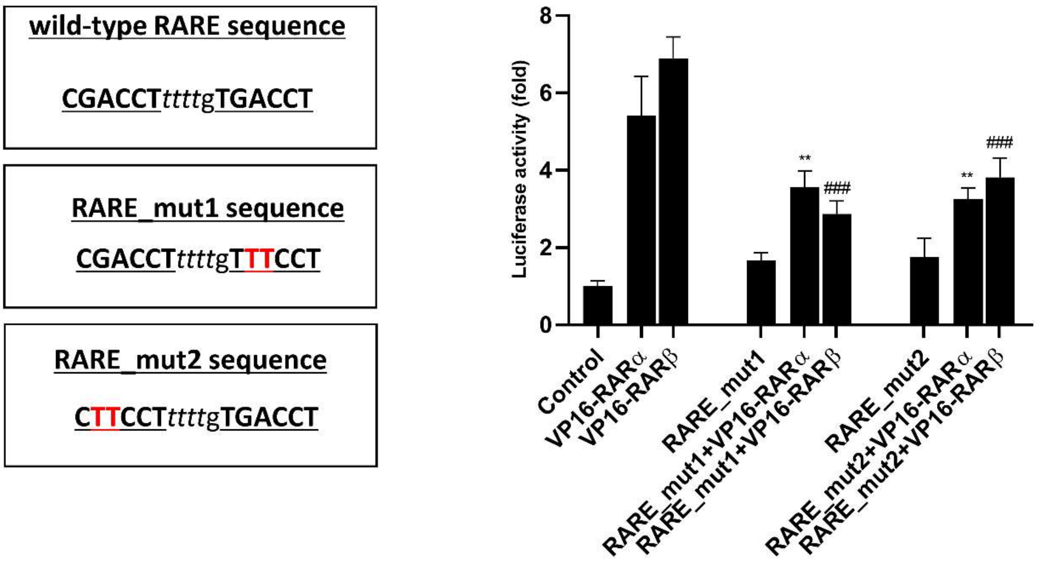

Figure S4. Effect of a RARE mutation in the *Prdm3* promoter on RARs stimulation. P19 cells were transfected with the *Prdm3* promoter carrying RARE wild-type, RARE_mut1, and RARE_mut2 sequences in combination with pcDNA3 (Control), RARα, or RARβ. Luciferase activity is shown as a fold change over control. Error bars are shown as mean ± S.E.M., n=4 per group. **p<0.01 compared to VP16-RARα and ### p<0.001 compared to VP16-RARβ.
